# Visual Servoed Autonomous Landing of an UAV on a Catamaran in a Marine Environment

**DOI:** 10.3390/s22093544

**Published:** 2022-05-06

**Authors:** Andrea Delbene, Marco Baglietto, Enrico Simetti

**Affiliations:** 1 Department of Informatics, Bioengineering, Robotics and Systems Engineering (DIBRIS), Università degli Studi di Genova, Via all’Opera Pia 13, 16145 Genoa, Italy; marco.baglietto@unige.it (M.B.); enrico.simetti@unige.it (E.S.); 2 Interuniversity Research Center on Integrated Systems for the Marine Environment, Via all’Opera Pia 13, 16145 Genoa, Italy

**Keywords:** UAV, ASV, splashproof quadrotor, vision system, state machine, autonomous landing, marine robotics, aerial robotics

## Abstract

This paper introduces a procedure for autonomous landing of a quadrotor on an unmanned surface vehicle in a marine environment. The relative pose and velocity of the vehicle with respect to the quadrotor are estimated using a combination of data coming from a vision system, which recognizes a set of AprilTags located on the vehicle itself, and an ultrasonic sensor, to achieve further robustness during the final landing phase. The considered software and hardware architecture is provided, and the details about the landing procedure are presented. Software-in-the-loop tests were performed as a validation step for the proposed algorithms; to recreate realistic conditions, the movements of the landing platform have been replicated from data of a test in a real marine environment. In order to provide further proof of the reliability of the vision system, a video sequence from a manual landing of a quadrotor on the surface vehicle in a real marine environment has been processed, and the results are presented.

## 1. Introduction

During the last years, unmanned aerial vehicles (UAVs) have been used in a wide variety of applications, such as in agriculture [1], civil protection [2], infrastructure, inspection and maintenance [3], and light shows [4]. Their uses are not limited to the Earth’s surface, but extend to space environments as well, where they could be used for ad hoc missions [5]. When considering applications in a marine environment, different types of robots can be involved. Generally, a set of underwater and surface vehicles allow the execution of complex tasks, such as monitoring wide areas or cooperative collaboration for a mutual goal. For instance, in [6], an autonomous robotic team composed of underwater and surface vehicles was considered for geotechnical survey purposes. By considering aerial agents also, a wider variety of missions can be designed, such as the protection and security of marine areas, or humanitarian search and rescue activities [7]. The landing of aerial agents requires ad hoc procedures [8], and when working in complex conditions, this is often performed by a human operator. In fact, in marine applications, the sea conditions could alter the pose of the landing target, and could determine the success or the failure of the landing procedure itself. Therefore, in fully autonomous missions, the landing maneuvers of an aerial agent must be robust to difficulties and reliable.

On the basis of the previous works [9,10], we aimed to provide an efficient, reliable, and modular solution to autonomously land a quadrotor on a catamaran in a marine environment.

### 1.1. Related Work

Performing an autonomous landing procedure on a platform is a complex task that requires several steps to be achieved. In outdoor scenarios, global navigation satellite system (GNSS) receivers usually provide the positions of the quadrotor and the catamaran. Still, the data coming from GNSS are not sufficient to perform an autonomous landing, due to their inaccuracy. Even if properly filtered [11], the accuracy and precision are not enough for such complex and precise maneuvers. Additionally, the catamaran is subjected to unpredictable oscillatory dynamics caused by sea behavior and weather conditions to which the quadrotor must be able to react. For these reasons, alternative approaches have to be taken into consideration. For instance, a video system would allow increasing the reliability and the performance of the landing procedure. The GNSS data are used by the quadrotor to move close to the position of the catamaran, and from there, a vision system gives the quadrotor the relative pose of the platform on the catamaran. Computer vision algorithms are extremely useful to close the loop when the landing platform is in an uncertain position or it is moving [12], since they allow directly estimating the horizontal and vertical tracking errors with respect to the target point, instead of providing its coordinates in an absolute frame. An interesting and efficient solution was proposed in [13], where an extended Kalman filter was developed to combine data coming from different sensors (inertial navigation, GNSS receiver, and visual sensor) to build a navigation system and perform a landing procedure. In [14], a solution composed of several LEDs and an “H” sign placed on the landing platform was proposed: the LEDs give the possibility to the unmanned aerial vehicle (UAV) of recognizing the platform from high altitudes by using an infrared camera, and the “H” sign helps the estimation of the center of the platform itself when the quadrotor is closer. Instead, in [15], helipads composed of different geometric shapes (a cross, a circle, and a square) were proposed, to test the designed vision-based autonomous landing algorithm. Experimental results have been achieved outside of the marine environment, with a mobile robot carrying a landing platform moving on the ground. Another solution for the estimation of the relative pose between the quadrotor and the landing platform is the one proposed in [16], where a specific marker composed of a series of concentric circles would allow the detection of the platform from close by.

A similar methodology is the one presented in [17], where a landing platform composed of several AprilTags [18,19] with different dimensions is introduced: the larger tags permit the detection from higher altitudes, and the smaller ones from lower altitudes. This allows the quadrotor to constantly track the landing platform while decreasing its altitude during the landing procedure. The choice of using AprilTags is mainly related to their versatility and robustness [20].

### 1.2. Contributions

The innovation of this paper with respect to the state of art is mainly the development of a set of software packages able to perform an autonomous landing procedure in a sea environment, where the catamaran is subject to wave-induced oscillations. The landing procedure is tackled by implementing a set of strategies, such as a preliminary positioning of the drone, platform searching, horizontal tracking to keep it aligned, and vertical compensation with respect to the landing platform. The behavior of the quadrotor during the whole landing procedure is handled by a finite state machine: a set of states and conditions that describe the actions the quadrotor has to perform, depending on the data coming from different sensors. An improved landing platform composed of more tags with respect to the past solution [9] has been designed. Simulations of autonomous landing have been performed in an environment composed by several tools, such as Gazebo (more info at: http://gazebosim.org/ last access: 30 March 2022), ROS2 (more info at: https://docs.ros.org/en/foxy/index.html, last access: 30 March 2022), and PX4 (more info at: https://px4.io/, last access: 30 March 2022), where the pose of the catamaran was replicated from data coming from sea tests involving only the catamaran itself so that the motion of the landing platform was realistic.

The proposed software architecture allows both the validation of the considered methodology via software-in-the-loop simulations and the integration of most of those components in the real hardware, as a preparation for tests in a real environment. To further validate the reliability and robustness of the onboard vision system, an onboard video captured from a manual flight landing of the quadrotor on the catamaran has been processed offline using the adopted vision system.

Therefore, with respect to the previous works [9,10], the contributions of the present manuscript are:

1. A new software architecture powered by the ROS2 middleware and designed specifically to be modular for both simulation tests and outdoor tests in a real marine environment;
2. Design and implementation of an improved landing state machine, with the addition of a new state and the introduction of a new procedure to synchronize the position of the quadrotor with the catamaran before the landing approach;
3. Simulations in a software-in-the-loop environment of a safe landing on a landing platform, whose movements were replicated from the telemetry of the catamaran recorded during outdoor tests in a marine environment;
4. Realization and integration on the catamaran of a new landing platform, with more tags to gain better robustness during the landing procedure;
5. Validation of the vision system using the recordings of a manual landing on the catamaran in a marine environment.

This paper is organized as follows: In Section 2, an overview of the whole system is proposed. In Section 3, the methodology of the proposed landing procedure is detailed, and in Section 4 the developed software/firmware architecture is presented. In Section 5, the main results obtained in flight emulation tests are shown. Finally, some conclusions are given in Section 6.

## 2. System Overview

The considered experimental system is composed mainly of two different agents, each one with unique characteristics and features.

### 2.1. Catamaran

**Figure 1:**
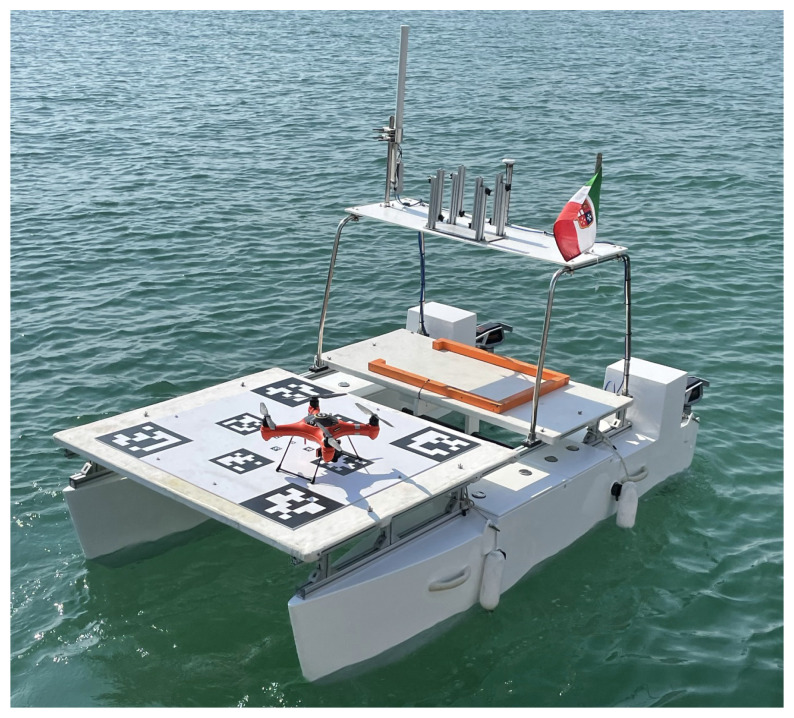
The ULISSE catamaran, equipped with the landing platform and the splash-proof quadrotor, deployed in one of the tests at sea.

The ULISSE autonomous surface vehicle (ASV), developed by the interuniversity research center for Integrated Systems for Marine Environment (ISME, University of Genova node), is a 3 m long and 1.8 m wide catamaran, constructed in fiberglass (see Figure 1). It was designed as a modular vehicle for various applications. When used for marine geotechnical surveys [21] or acting as an intelligent buoy for underwater vehicles, it carries a deck with an underwater mast with acoustic sensors. When used as a means to extend the action range of the aerial drones, the catamaran is equipped with a dedicated landing platform (as in Figure 1). In each hull of the catamaran, a compartment hosts batteries (around 3.2 kWh of energy each), the hardware architecture where the control software runs the *ROS2* middleware, and a wide range of sensors (GNSS receiver, gyroscopes, accelerometers, and a compass sensor) to collect ego-motion measurements. The catamaran is provided with a roll-bar where the GNSS antenna is located, along with a 5 GHz one for Wi-Fi communication. The catamaran is propelled by two Torqeedo Cruise 2R electric thrusters, with electrical power of 2 kW each, which offer high maneuverability of the vessel even at low speeds, making it very agile in cluttered areas.

### 2.2. Quadrotor

The chosen quadrotor model is the SwellPro Splash Drone 3 (more info at: https://swellpro.com/, last access: 30 March 2022), a drone that provides an external waterproof structure specifically designed for marine applications, along with various internal hardware components that allow performing manual flights. For the purpose of the realization of the proposed strategy, these components were substituted to robotize the agent itself. In particular, a Raspberry Pi Model B+ (more info at: https://www.raspberrypi.com/, last access: 30 March 2022) was embedded, along with a Raspicam v2, to allow onboard computations, and a Pixracer (more info at: https://docs.px4.io/master/en/flight_controller/pixracer.html, last access: 30 March 2022) autopilot system containing several embedded sensors, such as an accelerometer, a magnetometer, a gyroscope, and a barometer. The autopilot receives the setpoints computed by the algorithm running on the Raspberry Pi, and translates them into pulse width modulation (PWM) signals for the single motors of the quadrotor. The quadrotor is also endowed with a GNSS receiver. An ultrasonic sensor was included, as it is essential during the landing procedure, and so was a payload release mechanism actuated by a servo motor, to enable the quadrotor to carry out delivery tasks in a marine environment.

## 3. Methodology

The proposed landing solution is composed of different modules. Each one is detailed hereafter.

### 3.1. Perception and Pose Estimation

The relative pose of the quadrotor with respect to the landing platform is estimated by an onboard vision system that processes the video stream coming from the Raspicam. The platform is equipped with a set of visually distinguishable tags, each one being different from the others and characterized by a unique ID. The adopted vision system, named AprilTag, is an open-source, robust, and well documented tool [18,19] that allows 3D position and orientation computation of the considered tags with respect to the camera [20]. The use of a single tag does not guarantee its identification during the whole landing procedure; hence, the landing platform was equipped with 13 unique AprilTags, following a similar configuration as [17].

**Figure 2:**
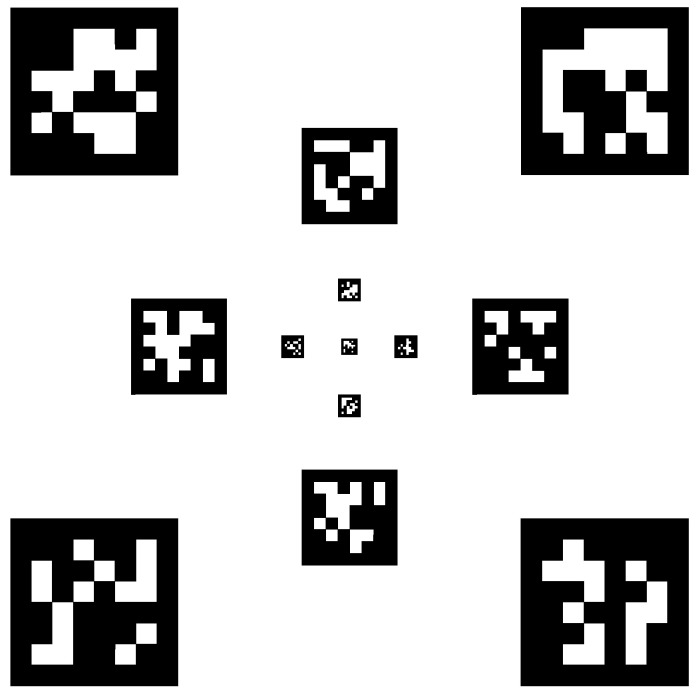
The set $S_{l}$ of AprilTags of different sizes as printed on the landing platform.

The tags, as shown in Figure 2, were placed in such a way as to guarantee visibility from different distances and robustness in the landing phase. The AprilTag markers on the outer edges are large, and thus easily recognizable at higher altitudes. The smaller internal ones play a crucial role in the final instants of the landing maneuver when the quadrotor is closer to the platform, improving safety and reliability.

The list of the detectable tags is represented by the set $S_{l}=\left\{1,\ldots ,13\right\}$. At each iteration of the vision system, the detected tags ID are stored in a subset $S_{d}\subseteq S_{l}$. For each detected tag ID $i\in S_{d}$, the vision system computes a transformation matrix ${}_{i}^{c}T$ that describes the position in the scene of the identified tag *i* with respect to the camera frame *c*. In order to compute the pose of the platform center with respect to the camera for each tag $i\in S_{l}$, a set of transformation matrices ${}_{p}^{i}T$—$i\in S_{l}$ describing the position of each tag with respect to the platform center—is calibrated and computed offline. Thus, a post-multiplication gives the needed transformation matrix:

(1) $${}_{p}^{c}T_{i}={}_{i}^{c}T\;{}_{p}^{i}T\;.$$

Theoretically, each detected tag gives equally correct information. However, to improve the quality of the estimation, these measures are merged and weighted by the areas ($a_{i}$) of each detected tag in the camera frame: $i\in S_{d}$. Thus, the weighted transformation matrix between the center of the platform and the camera on the quadrotor ${}_{p}^{c}T$ is obtained by:

(2) $${}_{p}^{c}T=\frac{1}{w}\sum_{i\in S_{d}}{}_{p}^{c}T_{i}a_{i}\;,$$

where *w* is the normalization term defined as:

(3) $$w=\sum_{i\in S_{d}}a_{i}\;.$$

**Figure 3:**
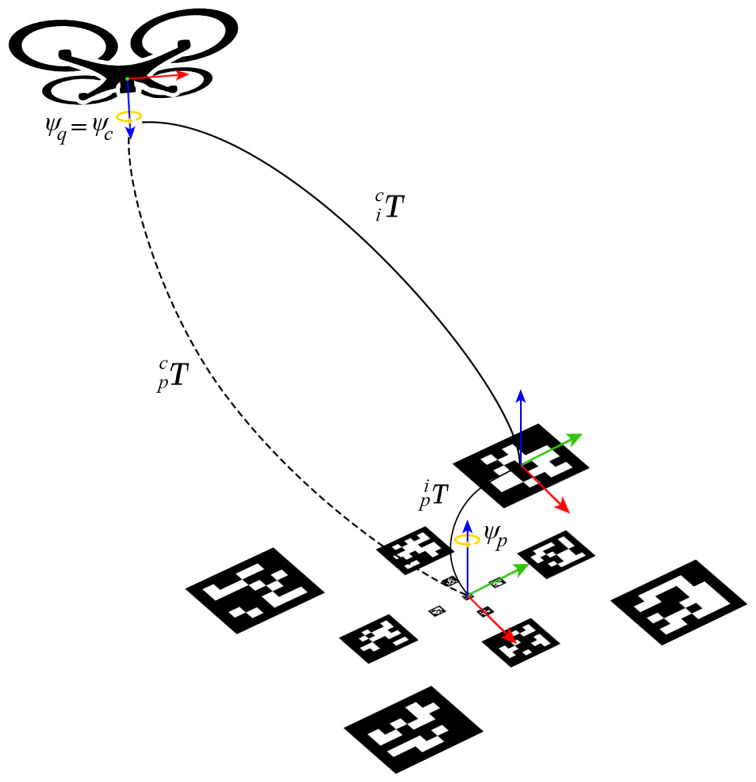
A representation of the different transformation matrices involved in the relative pose estimation between the UAV and the landing platform’s center.

The reference error is then transformed in the inertial frame, taking into account the quadrotor’s attitude (see Figure 3), and sent to the guidance controller, which generates the desired commands for the autopilot.

Still, depending on sea conditions, the vertical velocity of the landing platform could vary a lot, and the estimated vertical error using the vision system alone does not provide a reliable measure in the final instants of the landing phase. To increase robustness, an ultrasonic sensor pointing downward was installed on the quadrotor, providing distance information at a limited range. More precisely, the camera provides information at 20 fps (frames per second). The ultrasonic sensor provides information at 30 Hz, and at distances less than 0.75 m, provides more precise and reliable data. The distance data coming from the ultrasonic sensor are used to estimate the platform’s vertical velocity via a basic Kalman filter [9]. This information is merged with the estimated vertical error, as shown in Section 3.3.

### 3.2. Horizontal Platform Tracking

One of the tasks the quadrotor has to perform during the landing procedure is horizontal tracking of the platform, reducing the estimated horizontal error provided by the camera. By defining the horizontal positions of the quadrotor and the platform as $p_{q,xy}$ and $p_{l,xy}$, respectively, the horizontal position error is $e_{p,xy}=p_{l,xy}-p_{q,xy}$. A measure of this error is taken from the onboard vision system. Thus, a PI regulator is designed to produce position setpoints $p_{q,xy}^{*}$:

(4) $$p_{q,xy}^{*}=p_{q,xy}+K_{P}\;e_{p,xy}+K_{I}\int e_{p,xy}\;dt\;,$$

where $K_{P}$ and $K_{I}$ are the proportional and integral gains of the controller, respectively.

### 3.3. Vertical Platform Compensation

Once the quadrotor is at a certain distance from the landing platform, it needs to take care of the heave motions of the landing pad, induced by the waves. In this delicate phase, the altitude setpoints are generated to keep the relative velocity between quadrotor and catamaran to a specific value $v_{r,z}^{des}$. More in detail, a vertical target absolute velocity can be defined as:

(5) $$v_{q,z}^{des}=v_{r,z}^{des}+v_{l,z}\;,$$

where $v_{l,z}$ is obtained by:

(6) $$v_{l,z}=v_{q,z}-v_{r,z}\;,$$

and $v_{r,z}$ is the estimation of the relative vertical velocity obtained by the above mentioned Kalman filter. The vertical error velocity is defined as:

(7) $$e_{v,z}=v_{q,z}^{des}-v_{q,z}\;.$$

Thus, the desired vertical velocity is a proportional scale of (7) by a gain $K_{1}$:

(8) $$v_{q,z}^{*}=v_{q,z}+K_{1}\;e_{v,z}\;.$$

Finally, the altitude setpoints are generated as:

(9) $$p_{q,z}^{*}=p_{q,z}+K_{2}\;v_{q,z}^{*}\;,$$

where $p_{q,z}$ is the current altitude of the quadrotor, and $K_{2}$ is a scale gain. Vision system data are not used at this stage, since the ultrasonic sensor gives information at a higher frequency.

### 3.4. Finite State Machine

**Figure 4:**
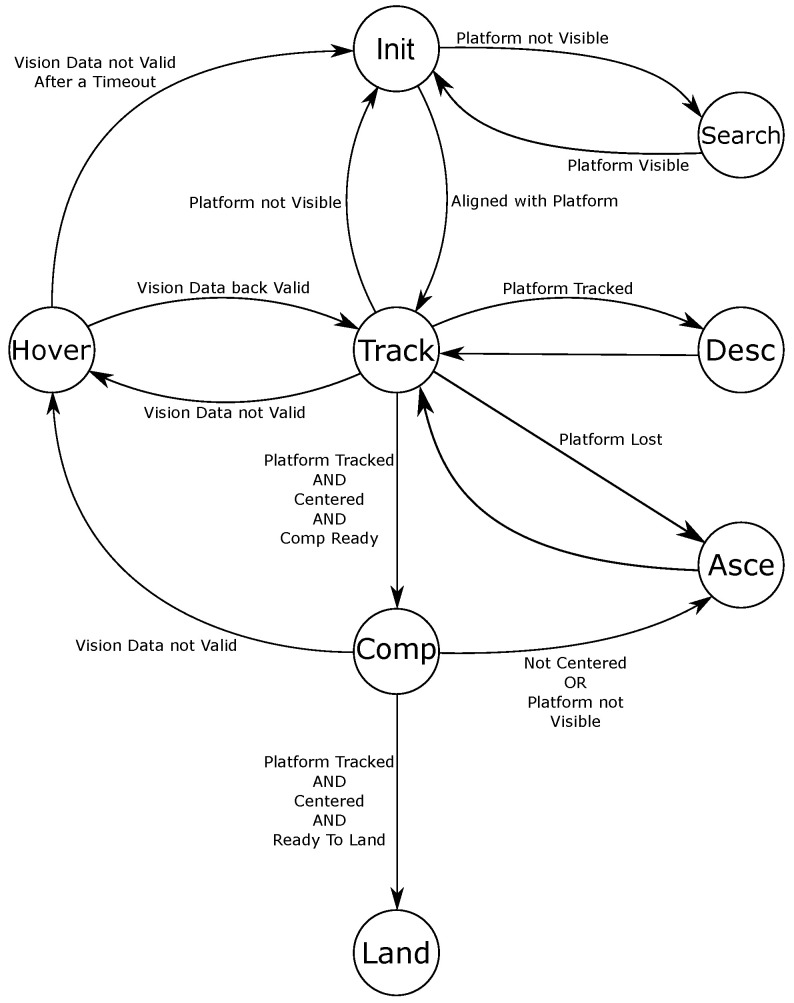
The diagram of the proposed finite state machine for the autonomous landing.

The landing phase is described by a series of connected states, whose transitions are handled by a finite state machine. The behavior of the quadrotor is described by eight states: initialization, searching, tracking, hovering, descending, ascending, compensation, and landing; Figure 4 describes how the states are linked. The transitions among them are triggered by boolean algebra operations.

Initially, the quadrotor performs the rendezvous with the catamaran. The latter sends a stream of its GNSS position to the former, which flies to reach it. The catamaran’s GNSS position is exploited only in the initial phase, as it can be imprecise, and would not guarantee a robust and reliable landing, especially in cases of signal loss. When the quadrotor reaches the area described by the received GNSS coordinates, the finite state machine starts, whose states are detailed in the following subsections.

#### 3.4.1. Initialization

This is the entry point of the procedure. In this state, the quadrotor reaches the starting altitude and starts to look for the landing platform. If the landing pad is not detected, the finite state machine changes the state to *searching*. Otherwise, the quadrotor places itself in a specific position and orientation with respect to the catamaran. The basic idea is to prevent landing from a position where the quadrotor could hit the roll-bar located on the stern side of the catamaran (see Figure 1).

**Figure 5:**
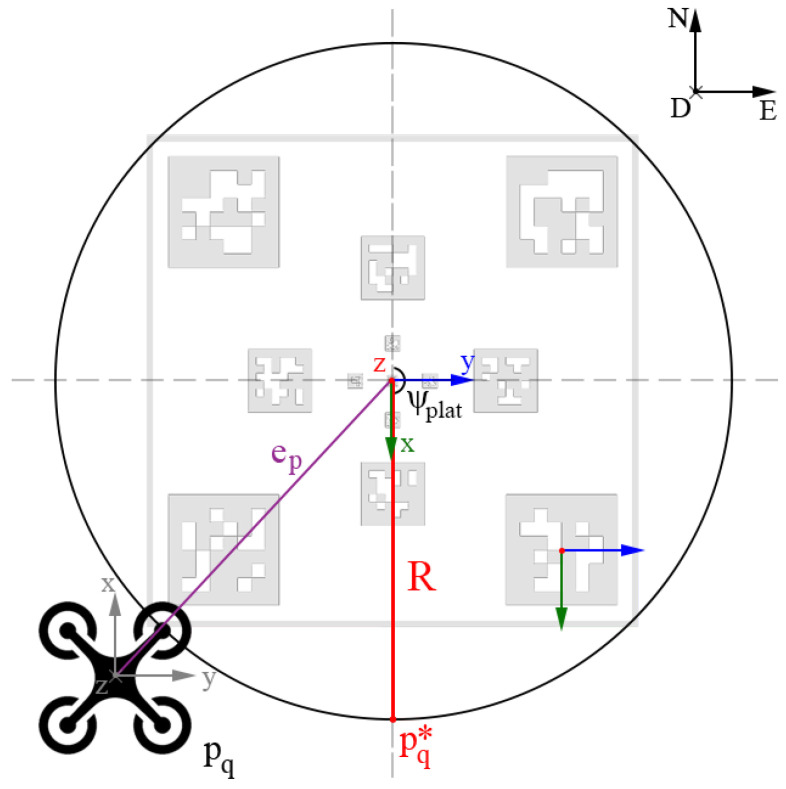
The generation of the initial relative position $p_{q}^{*}$ during the initialization phase. The quadrotor needs to place itself in front of the catamaran, and it does that by moving around the platform and placing at a certain distance from it.

For this purpose, as seen in Figure 5, the quadrotor is placed in front of the landing platform, at a certain distance from the center. In detail, the desired positions $p_{q,x}^{*}$ and $p_{q,y}^{*}$ are computed directly by:

(10) $$\begin{array}{ccc} p_{q,x}^{*} & = & p_{q,x}+e_{p,x}+R\sin\left(\psi_{l}\right) \end{array}$$

(11) $$\begin{array}{ccc} p_{q,y}^{*} & = & p_{q,y}+e_{p,y}+R\cos\left(\psi_{l}\right)\;, \end{array}$$

where $e_{p,x}$ and $e_{p,y}$ are the estimated horizontal error components (see Section 3.2), *R* is the desired fixed distance the quadrotor has to keep from the platform, and $\psi_{l}$ is the catamaran’s yaw angle. To prevent further complications in the quadrotor’s movements, its yaw is kept constant for the whole landing phase.

#### 3.4.2. Searching

If the quadrotor has no visual information about the position of the landing platform, it enters a state where it searches for it. To this end, the quadrotor reaches a predefined altitude and flies in circles increasing large in radius. In particular, by taking as the center point the quadrotor’s position at the initialization time of the searching phase $p_{q,x}\left(t_{0}\right),p_{q,y}\left(t_{0}\right)$, the desired position of the quadrotor is defined by:

(12) $$\begin{array}{ccc} p_{q,x}^{*}\left(t\right) & = & p_{q,x}\left(t_{0}\right)+R\cos\left(\frac{v_{s}}{R}\left(t-t_{0}\right)\right) \end{array}$$

(13) $$\begin{array}{ccc} p_{q,y}^{*}\left(t\right) & = & p_{q,y}\left(t_{0}\right)+R\sin\left(\frac{v_{s}}{R}\left(t-t_{0}\right)\right)\;, \end{array}$$

where *R* is the desired radius of the first circle and $v_{s}$ is the desired linear velocity to be tracked during this phase. The searching continues until the following condition is verified:

(14) $$\left(t-t_{0}\right)<\frac{2\pi R}{v_{s}}\;.$$

When this condition is no longer true, the parameters are updated: *R* is increased by 0.5 m so that the quadrotor inspects a new area while overlapping a part of the previous one, $t_{0}$ is set to the current value of *t* ($t_{0}=t$). By doing that, the condition returns true, and at the next iteration, the quadrotor starts a new circle, but with an increased radius. The structure of the second term of (14) makes sure the quadrotor starts a new circle in the exact instant when it finishes the first one.

Once the quadrotor detects the platform, the landing procedure begins, and the quadrotor goes back to the initialization state. This process guarantees the success of the action even if only the approximate position of the platform is known. If the quadrotor loses the platform when already landing, the searching state takes also into account the last computed vision error, so that the quadrotor centers itself in the last known position of the catamaran to restart the search.

#### 3.4.3. Tracking

When the quadrotor is correctly positioned with respect to the catamaran, the tracking state is triggered, handling the reduction of the vertical and horizontal error between the two agents. The descent of the quadrotor toward the catamaran becomes slanted; in particular, at the time instant $t_{h}$ indicating the moment this state starts, a slope between its current altitude and an altitude point $z_{max}$ (ideally, the maximum distance from the platform that allows the horizontal tracking of the smaller tags) is chosen. The *z* reference is computed using:

(15) $$z\left(t_{h}\right)=m\left(t_{h}\right)e_{p,x}\left(t_{h}\right)\;,$$

where

(16) $$m\left(t_{h}\right)=-\frac{p_{q,z}\left(t_{h}\right)-z_{max}}{R\sin(\psi_{l}\left(t_{h}\right))}\;.$$

#### 3.4.4. Hovering

This state handles the case when the vision data coming from the camera have not been updated for more than a second. In that case, the quadrotor is asked to keep its position for a certain period until the vision system is back online, sending again the required data. Then, once the feedback is restored, the quadrotor will resume its mission.

#### 3.4.5. Descending

This state gets triggered if the quadrotor is tracking the landing platform under a certain threshold and for a number of consecutive frames, but its current altitude is over the ideal horizontal maximum tracking altitude. The altitude waypoints are autonomously adjusted by being decreased by 0.1 m at each iteration.

#### 3.4.6. Ascending

This state gets triggered if the quadrotor has no visual contact with the landing platform for a number of consecutive frames, and its current altitude is below the ideal horizontal minimum tracking altitude. The altitude waypoints are autonomously adjusted by being increased by 0.1 m at each iteration.

#### 3.4.7. Compensation

When the quadrotor is under a certain vertical distance from the landing platform and it is centered with respect to it, the horizontal tracking (Section 3.2) and the vertical compensation (Section 3.3) tasks generate the position setpoints. In this state, to compensate for the catamaran’s oscillations, the vertical position setpoints are generated using the estimations coming from the Kalman filter and the equations reported in Section 3.3. The measures coming from the ultrasonic sensor are the only ones used, due to their higher-frequency updating.

#### 3.4.8. Landing

When the quadrotor is sufficiently close to the platform and the relative velocity between the two agents is under a certain threshold, the finite state machine enters the landing state, where the motors of the quadrotors are shut down, allowing it to land on the catamaran. Due to the criticality of this decision, the altitude and velocity thresholds have been set to very low values, to prevent crashes or mishaps.

## 4. Software Architecture

**Figure 6:**
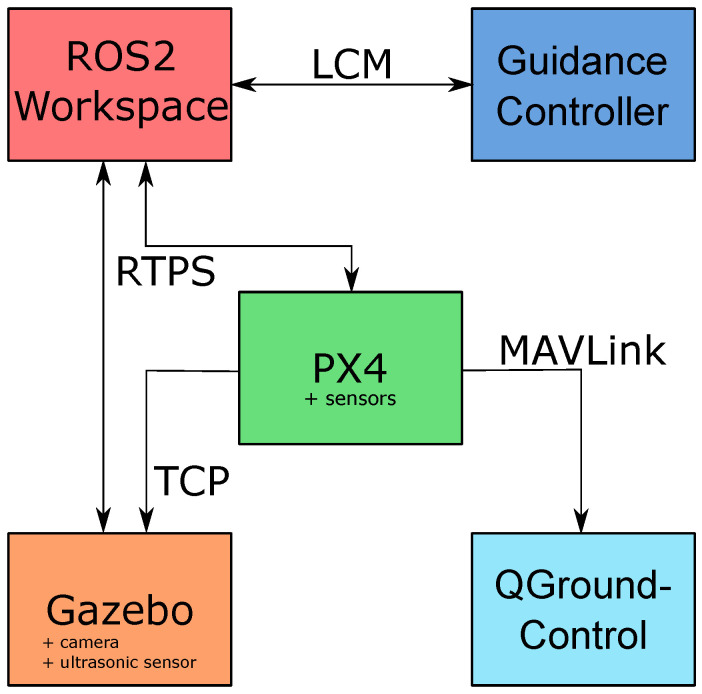
The main blocks composing the software architecture in the ROS2 simulation environment.

To process information coming from the various sensors and generate the setpoints necessary to fulfill the assigned tasks, the quadrotor needs to be equipped with a set of software tools communicating with each other, which were chosen strictly due to the hardware components that were installed on the quadrotor itself, presented in Section 2.2. An overview of the considered software architecture is presented in Figure 6.

### 4.1. Gazebo

Gazebo is software that makes it possible to simulate accurately and efficiently the dynamic behavior of populations of robots in complex environments. It offers an environment where the dynamics of the quarotor are approximately simulated. This was a necessary tool to test the developed algorithms, as an intermediate preparation for outdoor tests on real hardware.

### 4.2. PX4

PX4 is open-source flight control software for quadrotors and other unmanned vehicles. In this project, it is mainly used as a means to translate the pose setpoints coming from ROS2 nodes into PWM signals that are directly injected into the motors.

### 4.3. ROS2

ROS2 is a real-time version of the more common ROS (Robot Operating System), a set of open-access software libraries and tools for building robot applications. Its tools allow structuring the software in different modules interacting with each other through well defined message-based interfaces. In our study, ROS2 was used to create a workspace composed by a set of different modules, each one implementing a different feature: from packages collecting and processing the data coming from the camera and the ultrasonic sensor, to the ones handling the information transfer between the PX4 and the guidance controller. When performing a simulation, the sensors were replaced by their software counterparts implemented in Gazebo, and ROS2 nodes retrieved the data via RTPS (real-time publish–subscribe; see Section 4.6). Other software packages included the Kalman filter and the vision system described in Section 3.1. The vision system node is asynchronous and processes the compressed images coming from the camera whenever they are available (20 fps), requiring an average computational time of ∼7.0 ms. The measured maximum computational time was 29.86 ms, and the minimum was 1.56 ms; it depends on the number of visible markers. The node implementing the Kalman filter instead is synchronous with the ultrasonic sensor’s refresh rate of 30 ms, processing the coming data in an average of ∼3.0 ms; 4.7 ms maximally and 2.0 ms minimally.

### 4.4. Guidance Controller

The guidance controller is a software package that implements autonomous flight actions for the quadrotor. It is a modular open-source architecture written in C++, composed of several modules that communicate internally and externally via a communication protocol middleware named Lightweight Communications and Marshalling (LCM) [22]. The proposed architecture includes several macrotasks the quadrotor can perform—some of them simple, such as take-off from a point, navigation to point, and landing on a specific point; and others more complicated, designed for marine missions. The latter include searching and rescuing a shipwrecked person, landing on a platform, and criticality and failure handling. However, this paper mainly focuses on the autonomous landing on the catamaran.

### 4.5. QGroundControl

QGC (QGroundControl, more info at: http://qgroundcontrol.com/, last access: 30 March 2022) is software providing a graphical interface useful for monitoring a quadrotor’s status, full flight control, mission planning, and tuning of an autopilot system’s parameters.

### 4.6. RTPS

PX4-Fast RTPS Bridge (or more commonly RTPS, more info at: https://dev.px4.io/v1.11_noredirect/en/middleware/micrortps.html, last access: 30 March 2022) is a communication protocol that adds a real-time publish–subscribe (RTPS) interface to the PX4 Autopilot system, enabling the exchange messages between the various internal PX4 Autopilot components and ROS2 applications in real-time.

## 5. Emulation Results

### 5.1. Software-in-the-Loop Simulation

This section describes the results obtained with software-in-the-loop (SITL) tests performed with the help of the experimental architecture introduced in Section 4. To test the proposed methodology under realistic conditions, particularly as concerns the motion of the landing pad, we proceeded as follows. We first recorded to a log file of the telemetry (GNSS position and attitude) of the ULISSE catamaran executing a rendezvous with the quadrotor. The catamaran was directed toward a point and then instructed to hold its position. To do so, the catamaran positioned itself against the estimated direction of the current. However, note that the effects of waves and the fact that the catamaran is nonholonomic still induced a small lateral drift.

Then, we replicated the movement of the catamaran from the log file within the Gazebo environment and carried out several simulations of the quadrotor landing on it. Notice that, as the heave motion is not measurable in the real ULISSE ASV, we generated simulated motions using the Pierson–Moskowitz spectrum. Three different log files replicating the behavior of the catamaran were used. Over than 30 simulations have been performed; due to the similarity of the data among the logs, the catamaran’s behavior was replicated from the same log file for the majority of the tests. In a few of them, it happened that the quadrotor lost visual contact with the tags on the platform; in these cases, the quadrotor restarted the algorithm by resetting the state machine and approaching the landing pad again. Despite these setbacks, the quadrotor was able to land successfully on the platform in each simulation. For the sake of brevity, only the results of one simulations are reported hereafter.

**Figure 7:**
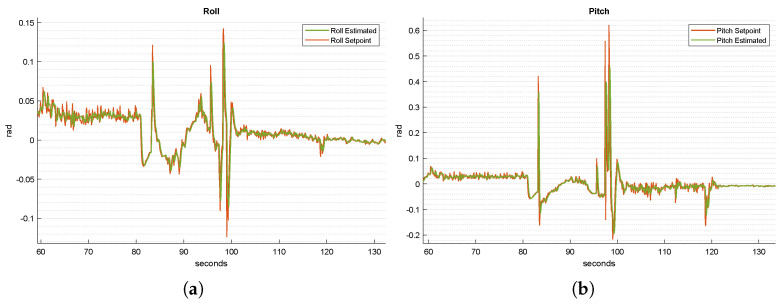
Time-wise behavior of the desired and estimated roll (**a**) and pitch (**b**) of the UAV during the landing procedure.

Let us begin by comparing the roll and pitch references and estimated values in Figure 7a and Figure 7b, respectively. From the figures, we can notice when the biggest adjustments in terms of position occurred from 82 to 98 s, when the procedure started with the searching state. Indeed, in this simulation, the UAV did not find the platform at the rendezvous point. Thus, the “searching” state was entered and the quadrotor started to move around in circles. From 98 to 118 s, the quadrotor placed itself in the front of the landing platform, to prepare for the landing. After then, before the quadrotor landed, the generated references varied slightly, because the effort needed to keep the alignment with the landing platform was minor. At around t=135 s, the quadrotor successfully landed on the platform.

The yaw reference was kept constant during the entire simulation, as it does not have a significant influence on the landing performance. Thus, its graph is omitted here.

**Figure 8:**
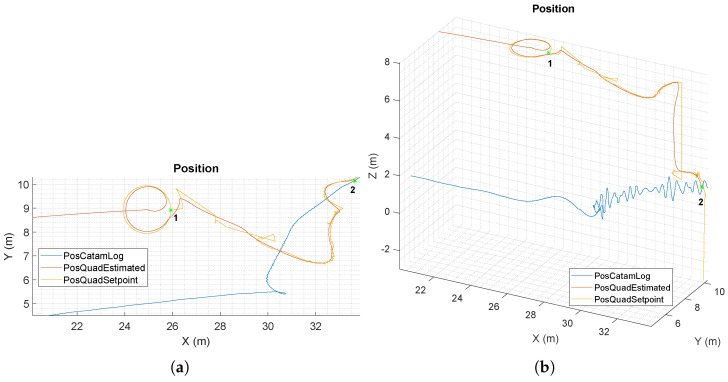
(**a**) Bird’s eye view of the trajectories of the UAV and of the ULISSE ASV during the simulation. 1 indicates the position at which the search radius changed, and 2 indicates where the final landing was accomplished. (**b**) A 3D representation.

The data about the positions of the quadrotor and the catamaran with respect to the starting point are depicted in Figure 8a,b. The first figure shows a bird’s eye view on the *x* and *y* dimensions only, and the second includes all the axes. The circular movements generated by Equations (12) and (13) during the searching state can be seen in the interval (x∈[24,26] m, y∈[8,10] m). The instant where the radius changed was point 1 (x=25.9 m, y=8.9 m). In this test, the quadrotor had to perform just one complete circle before seeing the platform at the start of the second one. The initialization phase came afterwards, where the quadrotor had to perform some adjustments to place itself on the bow side of the catamaran. It can be seen how the references slightly changed in the following period: this happened because the catamaran was sliding a bit while trying to keep its position against the sea current. More precisely, in this test the catamaran drifted laterally at an average velocity of ∼0.21 m/s. In order to find the maximum slide velocity the catamaran can have without compromising the landing of the quadrotor, several landing tests were performed in simulations with increasing drift velocity. The results of these simulations show that the quadrotor is able to successfully land at a drift velocity of up to 0.35 m/s, approximately. In Figure 8b, it can aksi be seen how the altitude of the catamaran had oscillating behavior in the final steps of the landing procedure. In fact, the catamaran’s heave movement is more affected by waves when it is not moving forwards. Still, the quadrotor managed to follow the catamaran for the entire period and accomplish the land at point 2 (x=33.6 m, y=10.1 m, z=2.0 m), showing the robustness of the proposed solution.

**Figure 9:**
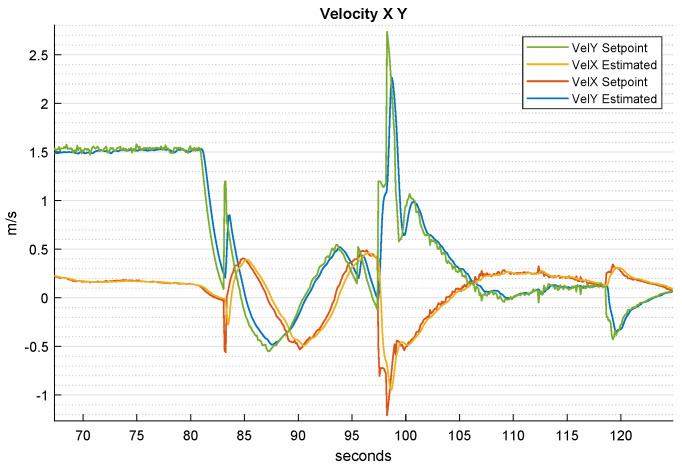
Time-wise behavior of the UAV’s *x* and *y* velocities and their setpoints during the whole landing procedure.

Figure 9 shows how the *x* and *y* axis velocities are generated starting from the pose references. In detail, during the searching phase from 82 to 98 s, the sum of the velocity components is approximately constant, corresponding to the parameter $v_{s}$ in Equations (12) and (13). This is not true when the quadrotor changes its circle’s radius, at 84 and 96 s. After that, the quadrotor makes visual contact with the landing platform; thus, the generated velocities are the ones needed to place the quadrotor appropriately for the landing.

**Figure 10:**
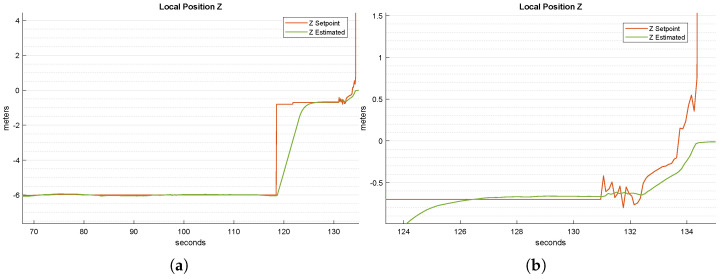
Time-wise behavior of the absolute *z* position and setpoint: (**a**) overall simulation; (**b**) a zoom of the compensation state. Notice that at t=134 s, a high setpoint was generated to force the UAV to freefall on top of the landing pad.

Figure 10a shows the quadrotor’s performances on the *z*-axis. As the inertial frame is a NED (north–east–down) frame, the sign of *z* is negative. Note the step-change reference at around t=118 s, stating the transition between the initialization and tracking state (Section 3.4.3) of the landing state machine. This brings the quadrotor to a prefixed minimum altitude from the landing platform, to trigger as soon as possible the switch to the compensation state. When the quadrotor is centered with respect to the landing platform and the Kalman filter taking data from the ultrasonic sensor gives reliable outputs, the state is switched to the compensation one, where the quadrotor compensates for the platform’s vertical motions (see Section 3.4.7).

Figure 10b shows a zoom of Figure 10a. The generated references change step by step, allowing the quadrotor to be as reactive as possible with respect to the platform’s oscillations. The peak at around t=134 s in Figure 10b shows the transition from the compensation to the landing state. At that time, the *z* reference is set to a value (20 m) well below the platform height above the sea, to force the UAV to set its motors to the minimum, making it free-fall directly onto the platform. Once the Autopilot’s firmware, PX4, has detected the landing, it automatically shuts off the motors in a few moments.

**Figure 11:**
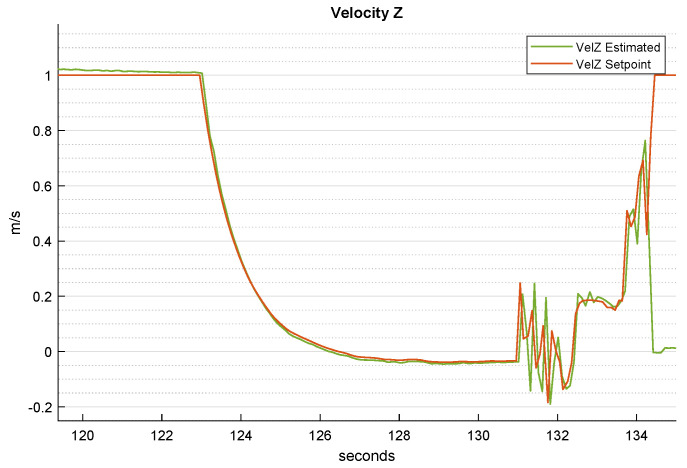
Time-wise behavior of the UAV *z* velocity and its setpoint during the whole landing procedure.

Figure 11 depicts the velocity on the *z* inertial axis. The generated references show the correlation among the *z* position setpoints. A full video of the simulation is available online and can be seen at: https://youtu.be/hILq4kUn9XY last access: 30 March 2022.

### 5.2. Vision System Validaton

To validate the pose estimation algorithm, an experimental trial where the quadrotor was manually controlled was conducted, and the output of the UAV’s camera was recorded at a resolution of 480p. Then, the video was replayed offline. Each frame was sent to the pose estimation algorithm. This experiment was not conceived to test the system under different lightning conditions. We selected a day with clear sky, as we wanted to test the material of the landing platform, which was selected to ensure a high level of opacity, to prevent light reflections on the markers. Note that in poor light conditions, for instance, during the evening, at night or on a cloudy day, the landing platform should be backlit to ensure reliable detection of the tags.

In the following, different images taken at various altitudes are presented, showing the detection performances of the proposed algorithm. In particular, the outputs of the pose estimation node, i.e., the detected nodes, are shown in magenta together with the tag identifier, on top of the same input image, to highlight the detection results.

**Figure 12:**
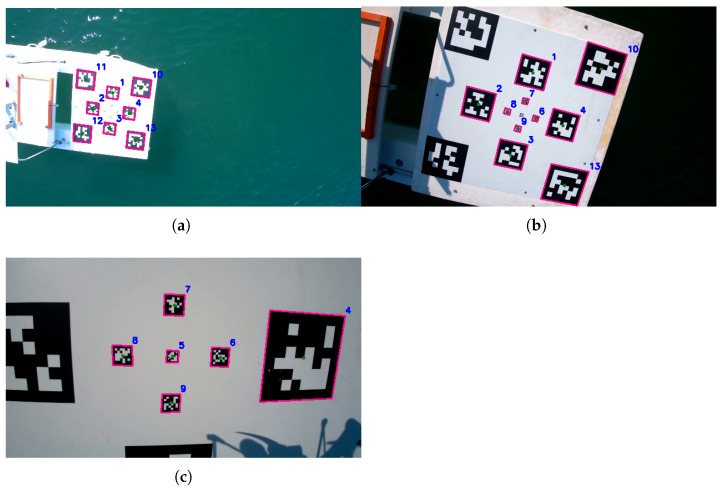
Detected tags at various altitudes: (**a**) initialization phase, high altitude; (**b**) tracking phase, medium altitude; (**c**) compensation phase, low altitude. Each detected tag is highlighted in magenta together with the tag identifier in blue.

Figure 12a depicts a situation where the distance bewtween quadrotor and catamaran is around 6–7 m, a case similar to the initialization phase. At that distance, in the best case, the detected tags are eight in number; the bigger tags play a fundamental role, offering reliability and robustness in the positioning of the quadrotor. The smaller ones are instead not recognizable yet. In Figure 12b, the quadrotor is landing on the catamaran. In a medium-distance case like this one, the number of tags detected is higher. In fact, the smaller ones become visible, except for the smallest one at the center, and some of the bigger ones can be out of camera range. Tag 11, on the upper-left corner, is a little outside of the image plane, and tag 12, in the lower-left corner, is obscured by the roll-bar’s shadow. Finally, Figure 12c shows the instants before the landing; there, the bigger tags are almost all not visible, whereas the smallest ones come into play to keep the quadrotor centered with respect to landing platform.

**Figure 13:**
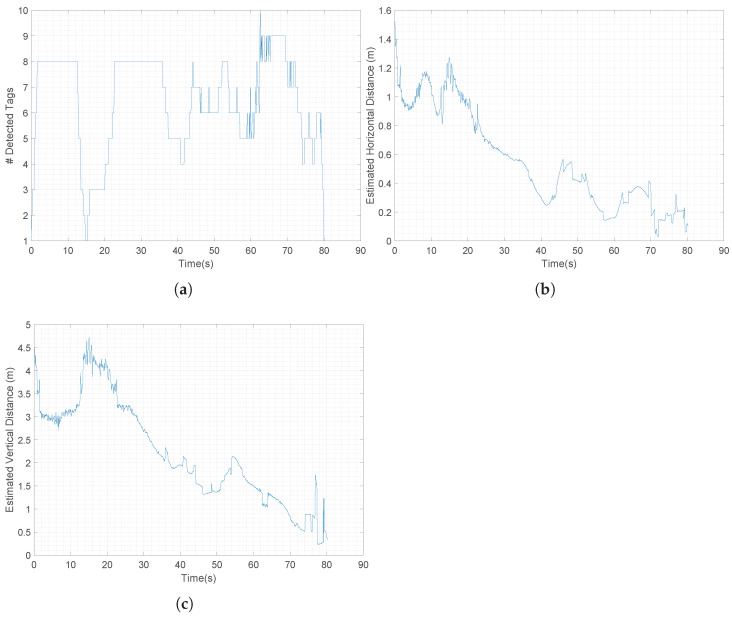
Evolution during manual landing experiment of: (**a**) number of tags detected; (**b**) horizontal error; (**c**) vertical error.

These last three graphs depict additional data from the vision system. In Figure 13a, the number of detected tags is shown, varying between 1 and 10 tags (corresponding to Figure 12b). It can be seen that the number of detected tags was always equal to or greater than one, assuring the continuity of the platform detection during the whole landing procedure, confirming the performances achieved in the simulation environment. The estimated horizontal (Figure 13b) and vertical (Figure 13c) errors with respect to the catamaran instead are pretty consistent with the quadrotor’s flight. A video of this emulation is available at https://youtu.be/iGNDCoQ2zaY last access: 30 March 2022. A further comparison of the vision system’s performance in the real test and in simulations has been performed in terms of marker detectability: it has been noticed that the smaller markers (IDs 6, 7, 8, 9) became visible when the relative distance of quadrotor–landing platform was approximately under 1.5 m both in simulations and in the real tests. Instead, the bigger ones (IDs 10, 11, 12, 13) could be detected at distances up to 9.0 m in the simulations.

## 6. Conclusions

In this work, a procedure for the autonomous landing of a quadrotor on a catamaran has been presented. The objective was to propose a specific, reliable, and robust architecture composed of different modules, adaptable to both simulations and tests in a real environment. A vision system relying on AprilTags has been proposed to recognize the landing platform; several tags have been placed on the platform itself to always assure recognizability during the entire procedure.

A finite state machine handles the landing procedure. It is composed of different states, each one describing a specific behavior the quadrotor has to engage.

The validity of the proposed methodology has been shown in a simulation environment. To test the landing procedure with realistic motions of the landing pad, the motion of the ULISSE ASV was recorded at sea and then replayed within the Gazebo environment. The proposed vision system was further verified using a pre-recorded video of a landing performed under direct teleoperation of the quadrotor.

## Data Availability

Not applicable.

## References

[1] Tsouros D.C., Bibi S., Sarigiannidis P.G. (2019). A Review on UAV-Based Applications for Precision Agriculture. Information.

[2] Boccardo P., Chiabrando F., Dutto F., Tonolo F.G., Lingua A. (2015). UAV Deployment Exercise for Mapping Purposes: Evaluation of Emergency Response Applications. Sensors.

[3] Jordan S., Moore J., Hovet S., Box J., Perry J., Kirsche K., Lewis D., Tse Z.T.H. (2018). State-of-the-art technologies for UAV inspections. IET Radar Sonar Navig..

[4] Huang J., Tian G., Zhang J., Chen Y. (2021). On Unmanned Aerial Vehicles Light Show Systems: Algorithms, Software and Hardware. Appl. Sci..

[5] Sharma M., Gupta A., Gupta S.K., Alsamhi S.H., Shvetsov A.V. (2022). Survey on Unmanned Aerial Vehicle for Mars Exploration: Deployment Use Case. Drones.

[6] Simetti E., Indiveri G., Pascoal A.M. (2021). WiMUST: A cooperative marine robotic system for autonomous geotechnical surveys. J. Field Robot..

[7] Casalino G., Allotta B., Antonelli G., Caiti A., Conte G., Indiveri G., Melchiorri C., Simetti E. ISME research trends: Marine robotics for emergencies at sea. Proceedings of the 2016 OCEANS.

[8] Kong W., Zhou D., Zhang D., Zhang J. Vision-based autonomous landing system for unmanned aerial vehicle: A survey. Proceedings of the 2014 International Conference on Multisensor Fusion and Information Integration for Intelligent Systems (MFI).

[9] Bastianelli Naticchi N., Baglietto M., Sperindé A., Simetti E., Casalino G. Visual Servoed Autonomous Landing on a Surface Vessel. Proceedings of the OCEANS 2019 MTS/IEEE.

[10] Nisticó A., Baglietto M., Simetti E., Casalino G., Sperindé A. Marea project: UAV landing procedure on a moving and floating platform. Proceedings of the OCEANS 2017.

[11] Abdelkrim N., Aouf N., Tsourdos A., White B. Robust nonlinear filtering for INS/GPS UAV localization. Proceedings of the 2008 16 th Mediterranean Conference on Control and Automation.

[12] Gautam A., Sujit P.B., Saripalli S. A survey of autonomous landing techniques for UAVs. Proceedings of the 2014 International Conference on Unmanned Aircraft Systems (ICUAS).

[13] Yang X., Mejias L., Garratt M. Multi sensor data fusion for UAV navigation during landing operations. Proceedings of the 2011 Australian Conference on Robotics and Automation (ACRA).

[14] Wang L., Bai X. (2018). Quadrotor Autonomous Approaching and Landing on a Vessel Deck. J. Intell. Robot. Syst..

[15] Falanga D., Zanchettin A., Simovic A., Delmerico J., Scaramuzza D. Vision-based autonomous quadrotor landing on a moving platform. Proceedings of the 2017 IEEE International Symposium on Safety, Security and Rescue Robotics (SSRR).

[16] Verbandt M., Theys B., Schutter J.D. Robust marker-tracking system for vision-based autonomous landing of VTOL UAVs. Proceedings of the 2014 International Micro Air Vehicle Conference and Competition (IMAV).

[17] Araar O., Aouf N., Vitanov I. (2017). Vision Based Autonomous Landing of Multirotor UAV on Moving Platform. J. Intell. Robot. Syst..

[18] Olson E., Strom J., Morton R., Richardson A., Ranganathan P., Goeddel R., Bulic M., Crossman J., Marinier R. (2012). Progress toward multi-robot reconnaissance and the MAGIC 2010 competition. J. Field Robot..

[19] Mersch D., Crespi A., Keller L. (2013). Tracking Individuals Shows Spatial Fidelity Is a Key Regulator of Ant Social Organization. Science.

[20] Olson E. AprilTag: A robust and flexible visual fiducial system. Proceedings of the 2011 IEEE International Conference on Robotics and Automation.

[21] Simetti E., Indiveri G. (2022). Control oriented modeling of a twin thruster autonomous surface vehicle. Ocean Eng..

[22] Huang A., Olson E., Moore D.C. Lcm: Lightweight communications and marshalling. Proceedings of the 2010 IEEE/RSJ International Conference on Intelligent Robots and Systems.

